# Different efficacy of EGFR tyrosine kinase inhibitors and prognosis in patients with subtypes of EGFR-mutated advanced non-small cell lung cancer: a meta-analysis

**DOI:** 10.1007/s00432-014-1709-0

**Published:** 2014-06-08

**Authors:** Huan Wang, Jing Huang, Xiaojin Yu, Shuhua Han, Xing Yan, Siqing Sun, Xiaoli Zhu

**Affiliations:** 1 Medical School of Southeast University, Nanjing, China; 2 Zhongda Hospital of Southeast University, Dingjiaqiao 87, Gulou District, Nanjing, 210009 China; 3 Public Health School of Southeast University, Nanjing, China

**Keywords:** NSCLC, EGFR, EGFR TKIs, Meta-analysis

## Abstract

**Background:**

Nearly 85 % of lung-cancer-specific epidermal growth factor receptor (EGFR) sensitive mutations comprise a substitution at position 858 (21L858R) and deletion mutants in exon 19 (19del). The aim of this study was to assess the role of EGFR mutation subtypes in predicting the efficacy of EGFR tyrosine kinase inhibitors (EGFR TKIs) and the prognosis of patients with advanced non-small cell lung cancer (NSCLC).

**Method:**

We systematically searched for eligible articles investigating the association between EGFR mutation subtypes and the efficacy of EGFR TKIs and the prognosis of patients with NSCLC. The summary risk ratio (RR) and mean difference (MD) were calculated using meta-analysis. In addition, we used variance analysis for the progression-free survival data (PFS) and used the rank sum test for the overall survival data.

**Results:**

We identified 22 eligible trials involving 1,082 patients. The objective response rate of the 19del mutation group was significantly higher than the 21L858R mutation group (RR 1.23; 95 % CI 1.12–1.36; *P* < 0.0001). The PFS (MD 3.55; 95 % CI 0.90–6.20; *P* = 0.009; MD 2.57; 95 % CI 0.51–4.62; *P* = 0.01) and overall survival (OS) (MD 10.52; 95 % CI 5.10–15.93; *P* = 0.0001) of the 19del mutation group were significantly longer than the 21L858R mutation group; the same results were observed in the variance analysis and rank sum test.

**Conclusion:**

The 19del mutation may be a more efficient clinical marker for predicting the response of patients with NSCLC to EGFR TKIs. Furthermore, patients with the 19del mutation have both a longer PFS and OS. The 19del mutation is also the prognostic factor for patients with NSCLC.

## Introduction

The small-molecule epidermal growth factor receptor (EGFR) tyrosine kinase inhibitors (TKIs), such as erlotinib and gefitinib, have been widely used in the treatment of advanced non-small cell lung cancer (NSCLC) (NCCN 2014). With the IDEAL1 study proving the efficacy of gefitinib for advanced NSCLC patients as a second-line treatment in 2003 (Fukuoka et al. 2003), and the BR.21 study indicating the efficacy of erlotinib for advanced NSCLC patients with chemotherapy failure in 2005 (Tsao et al. 2005), the development of EGFR TKIs was a milestone in NSCLC treatment.

Many studies have indicated that EGFR sensitive mutations are associated with a better efficacy of EGFR TKIs. Mokita’s analysis of seven clinical trials showed that patients with EGFR mutations responded better to EGFR TKIs, having a higher objective response rate (ORR) (79.3 %) and longer progression-free survival (PFS) (10.7 months) (Morita et al. 2009). A meta-analysis of phase III clinical trails (Gao et al. 2012) (including IPASS/OPTIMAL/EURTAC.) also showed that EGFR TKIs in NSCLC patients with EGFR sensitive mutations brought the ORR up as high as 66.6 %, with a median progression-free survival (mPFS) of 9.5 months and a median overall survival (mOS) of 30.5 months, as the first-line treatment. A recent pooled analysis (Paz-Ares et al. 2010) of 54 studies demonstrated that EGFR mutations predict response to EGFR TKIs, with erlotinib and gefitinib treatment showing significantly higher mPFS times of 13.2 and 9.8 months, respectively, when compared with only 5.9 months for chemotherapy treatment alone.

EGFR mutations occur almost exclusively within exons 18–21 of the gene, which encodes the amino lobe and part of the carboxy lobe of the receptor. Nearly 85 % of lung-cancer-specific EGFR mutations comprise a leucine-to-arginine substitution at position 858 (21L858R) and a deletion mutation in exon 19 that affects the conserved sequence LREA (19del) (Rosell et al. 2009). Is there any difference in response to EGFR TKIs between the two common types of patients with EGFR mutations? Several clinical studies suggested that patients with the exon 19 deletion had a better response rate, and a significantly longer PFS and OS than patients with L858R (Mitsudomi et al. 2010; Fukuoka et al. 2011). However, several other studies indicated that the difference in EGFR TKI efficacy between the two subtypes of EGFR mutations is not statistically significant (Cappuzzo et al. 2007).

We performed this meta-analysis, to compare the difference in EGFR TKI efficacy and determine the prognosis of advanced NSCLC patients with the two common subtypes of EGFR mutations (exon 19del and exon L858R), aiming to more accurately predict the response to EGFR TKIs and the prognosis for advanced NSCLC patients with activating EGFR mutations.

## Methods

### Search strategy

We performed a computerized search of Pubmed, Embase, and the Chinese National Knowledge Infrastructure (CNKI) database, we also manually searched the conference proceedings of the American Society of Clinical Oncology (ASCO), the European Society of Medical Oncology (ESMO) and the International Association for the Study of Lung Cancer from the year of 2000–2013 for relevant clinical trials. Reference lists from studies were also hand searched for this review. The following keywords were used: “NSCLC” or “non-small cell lung cancer” and “gefitinib or erlotinib” and “response.” There was no language restriction.

### Inclusion criteria

1. All prospective and retrospective studies were eligible for the study pool, only the most recent publication results from the same studies were included.
2. The patients who had a diagnosis of advanced NSCLC with stage III–IV and the patients harboring activating EGFR mutations (either exon 19 deletion or L858R in exon 21).
3. All patients received EGFR TKIs (gefitinib or erlotinib) for monotherapy, first line or otherwise.
4. All the included patients were evaluated by their treatment response, PFS and OS. Response Evaluation Criteria on Solid Tumors (RECIST) criteria were used to define response, with “complete response” or “partial response” classified as “response,” and “stable” with no change, or “progressive”.

### Exclusion criteria

1. Review, in vitro and animal experiments.
2. The objects of studies were not NSCLC patients.
3. The study of EFGR mutation in peripheral blood.
4. EGFR TKIs were used as maintenance or adjuvant therapy, or as sequential treatment with chemotherapy.

### Quality assessment and data extraction

The randomized controlled studies were assessed in strict accordance with the Jadad scale (Jadad et al. 1996), and the other studies were assessed in terms of the Newcastle-Ottawa Scale (NOS) (Wells et al. 2012; Stang 2010). To avoid evaluation deviation, two reviewers performed the assessment independently and discussed in order to reach an accordance when the information was unsatisfactory.

The following data were collected from each study: (1) general information: first author’s surname, publication time, source of the trial and the research type. (2) Features of the literature: clinical stage (AJCC staging), total numbers of the EGFR mutation types. (3) Evaluation indexes: ORR, PFS or TTP, and OS (Parmar et al. 1998).

### Data analysis and statistical methods

The meta-analysis of risk ratios (RRs) for the objective responses to treatment and the meta-analysis of mean differences (MDs) for PFS and OS were calculated using Review Manager (RevMan), version 5.2 (The Nordic Cochrane Centre, Copenhagen, Denmark) (The Cochrane Collaboration 2008).

A statistical test with a *P* value <0.05 was considered significant. RR > 1 reflects a better overall response rate in the exon 19del arm, MD > 1 reflects fewer deaths or progression in the exon 19del arm and vice versa. In each meta-analysis, Cochran’s Q statistic and *I* ^2^ statistics were calculated first in order to assess the heterogeneity among the proportions of the included trials. In case the *P* value was found to be <0.05, the assumption of homogeneity was deemed invalid and a random effects model was reported. Otherwise, a fixed effects model was reported. All *P* values were two sided. All CIs had a two-sided probability coverage of 95 %. If the study provided medians and interquartile ranges instead of means and SDs, we inputed the means and SDs as described by Hozo et al. (2005). When the literature failed to provide the standard deviation, we estimated it with the maximum and minimum values of similar studies from the included literature.

At the same time, we performed variance analyses for the progression-free survival of both exon 19del and exon 21L858R arms and used Mann–Whitney *U* test for the overall survival of both exon 19del and exon 21L858R arms, with a *P* value <0.05 considered to be significant. The calculations were performed by SPSS 18.0 (SPSS Inc., USA).

## Results

### Study identification

**Fig. 1:**
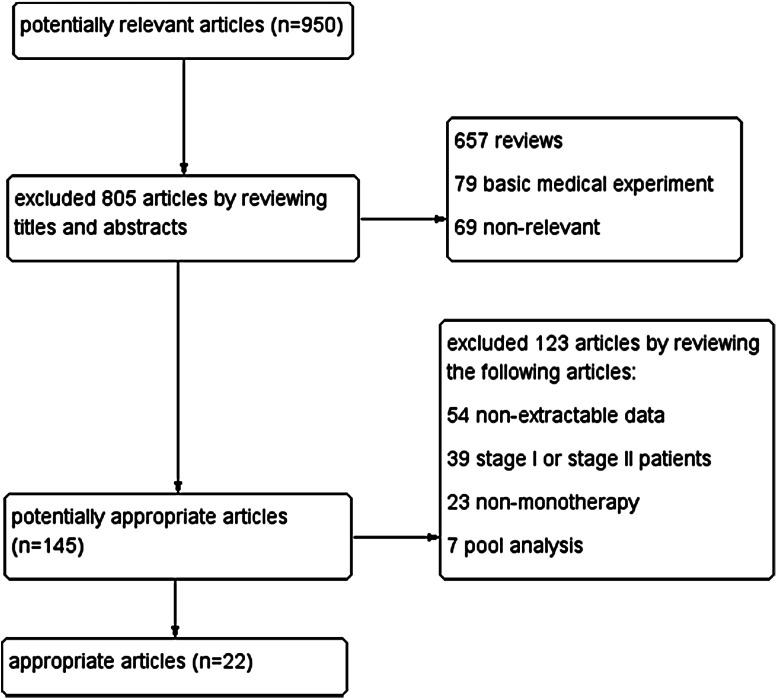
Electronic search flow chart

As shown in the NSCLC flow chart (Fig. 1), our initial search yielded 950 potentially relevant published articles. A review of the titles and abstracts of these articles resulted in 145 promising articles. These remaining 145 articles were selected for analysis and evaluated in greater detail by reviewing the full articles. After exclusion of the studies that did not meet the inclusion criteria, 22 studies with 1,082 patients were included in the meta-analysis, 593 patients with the exon 19del mutation and 489 patients with the exon 21L858R mutation. The characteristics of the eligible studies are summarized in Table 1.

**Table 1:** Characteristics of the 22 trials included in the meta-analysis

First author (year)	Race	Clinical stage	Number		Treat line	Treatment			Type of article	Score
			19 del	21 L858R		ORR	PFS	OS		
Sunaga et al. (2007)	A	III-IV	16	3	1	✓			Pro	6
Yoshida et al. (2007)	A	IIIB-IV	8	13	Mix	✓	✓		Pro	8
Sequist et al. (2008)	C	IIIB-IV	17	9	1	✓			Pro	7
Yang et al. (2008)	A	IIIB-IV	20	23	1	✓	✓		Pro	6
Asahina et al. (2006)	A	IIIB-IV	13	3	Mix	✓			Pro	7
Fukuoka et al. (2011)	A	IIIB-IV	66	64	1	✓			RCT	5
Maemondo et al. (2010)	C	IIIB-IV	58	42	1	✓	✓		RCT	4
Jackman et al. (2006)	C	IIIB-IV	22	10	1	✓	✓		Pro	7
Sutani et al. (2006)	A	III-IV	20	7	Mix	✓			Pro	8
Cappuzzo et al. (2007)	C	IIIB-IV	17	7	≥2	✓	✓		Pro	6
Tamura et al. (2008)	A	III-IV	14	17	Mix	✓			Pro	7
Sugio et al. (2009)	A	IIIB-IV	7	10	1	✓			Pro	6
Takano et al. (2007)	A	IIIB-IV	49	36	Mix	✓	✓		Retro	6
Chou et al. (2005)	A	IIIB-IV	11	12	Mix	✓			Retro	5
Ichihara et al. (2007)	A	IIIB-IV	16	14	Mix	✓			Retro	7
Pallis et al. (2007)	C	IIIB-IV	6	3	≥2	✓	✓		Retro	5
Wu (2010)	A	IIIB-IV	34	23	≥2	✓	✓	✓	Retro	5
Xu et al. (2009)	A	IIIB-IV	11	6	≥2	✓	✓	✓	Retro	7
Hirsch et al. (2007)	C	IIIB-IV	11	31	Mix	✓	✓	✓	Retro	7
马 (2013)	A	IIIB-IV	29	36	Mix		✓		Retro	4
Mitsudomi et al. (2010)	A	IIIB-IV	87	85	1		✓		RCT	5
Rosell et al. (2009)	C	IIIB-IV	57	29	1		✓		Pro	8

The quality of the randomized studies was assessed by using the Jadad Scale with 0–3 (low quality) and 4–5 (high quality). The quality of the nonrandomized studies was assessed by using the Newcastle-Ottawa Scale (NOS) with 0–5 (low quality) and 6–9 (high quality)

*Ref.* reference; *C* Caucasian; *A* Asian; *pro* prospective; *retro* retrospective; Treat line: 1 = only first-line EGFR TKI therapy; ≥2 = second-line therapy, third-line EGFR TKI therapy, ect.; mix = any one treat line EGFR TKI therapy

### Response rate

Data for the ORR were available in 19 trials, with 12 prospective trials and 7 retrospective trials, respectively. The *I* ^2^ statistic in the fixed effects model did not demonstrate significant heterogeneity in the results (*I* ^2^ 37 %; *P* = 0.06); the fixed effects model was used to pool the risk ratio for the included studies.

**Fig. 2:**
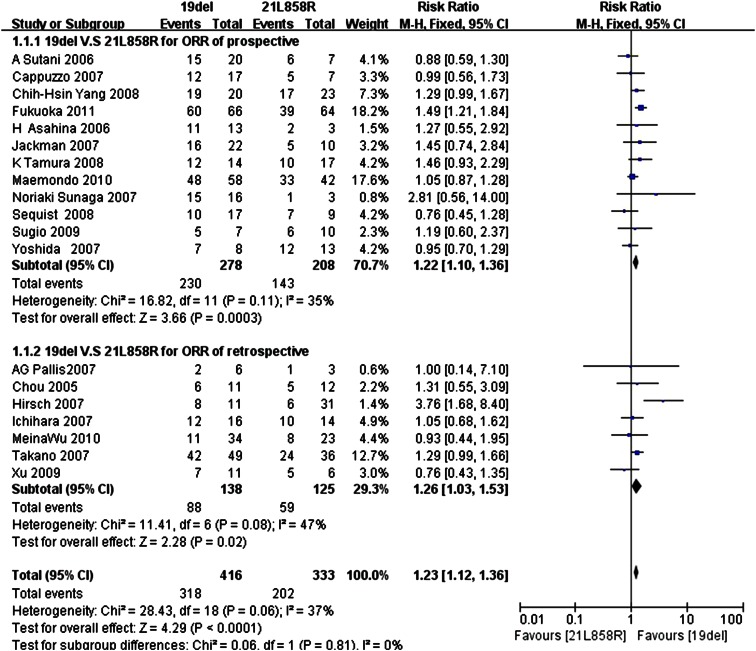
Forest plot of ORR among patients with EGFR 19del or 21L858R mutations. The *squares* and *horizontal lines* correspond to the study-specific RR and 95 % CI. The area of the squares reflects the weight. The *diamond* represents the summary RR and the 95 % CI

Analysis of this data demonstrated that the ORR of the exon 19del group was 76.44 % (318/416), which was higher than the 60.66 % (202/333) found for the exon 21L858R group,and the difference was statistically significant. (RR = 1.23; 95 % CI 1.12–1.36; *P* < 0.0001) (Fig. 2).

Furthermore, subgroup analysis revealed that the ORR was significantly different between the two groups (prospective trials: RR = 1.22, 95 % CI 1.10–1.36; retrospective trials: RR = 1.26, 95 % CI 1.03–1.53).

### Progression-free survival

Data for the PFS were available in thirteen trials, with seven prospective trials and six retrospective trials, respectively. The standard deviation could be obtained in five trials; the range of the standard deviation values was 5.59–14.7 in prospective trials and 2.97–9.94 in retrospective trials.

**Fig. 3:**
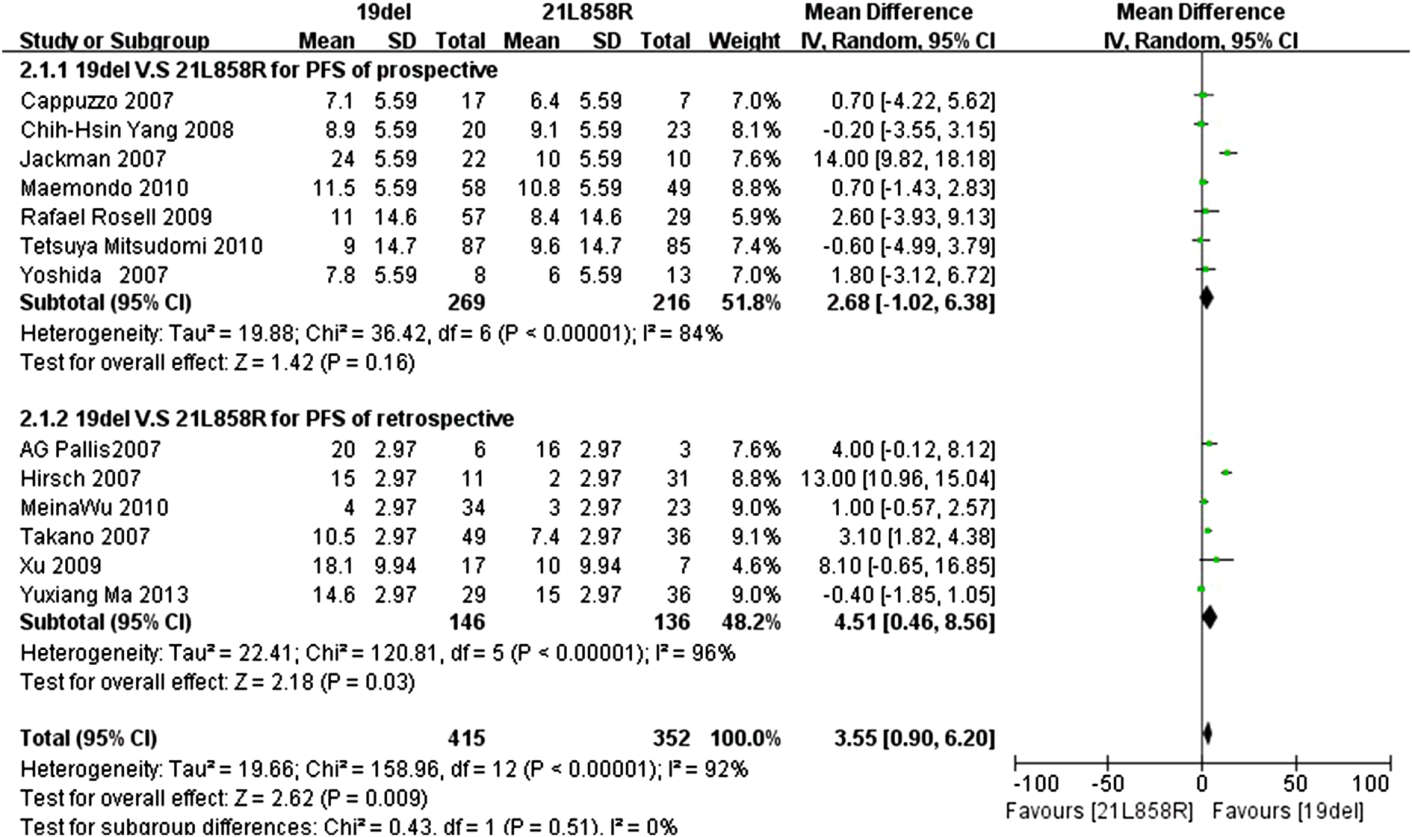
Forest plot of PFS among patients with EGFR 19del or 21L858R mutations with minimum SD. The *squares* and *horizontal lines* correspond to the study-specific MD and 95 % CI. The area of the *squares* reflects the weight. The *diamond* represents the summary RR and 95 % CI

We set the standard deviation to the minimum value of 5.59 for the other four prospective trials, and the minimum value 2.97 was used for the other four retrospective trials. Because the *I* ^2^ statistic in the fixed effects model demonstrated statistically significant heterogeneity in the results (*I* ^2^ 92 %; *P* < 0.0001), a random effects model was used to pool the mean difference for the included studies. As Fig. 3 demonstrates, patients with exon 19del mutation had a statistically significant longer PFS than patients with exon 21L858R mutations (MD 3.55; 95 % CI 0.90–6.20; *P* = 0.009). As the subgroup analysis shows, in prospective trials, subgroup patients with the exon 19del mutation had a longer PFS than patients with the exon 21L858R mutations, but the difference was not statistically significant (MD 2.68; 95 % CI −1.02 to 6.38; *P* = 0.16; *I* ^2^ = 84 %; random effects model). However, patients with the exon 19del mutation had a statistically significant longer PFS than patients with the exon 21L858R mutations in the retrospective trials subgroup (MD 4.51; 95 % CI 0.46–8.56; *P* = 0.03; *I* ^2^ = 96 %, random effects model).

**Fig. 4:**
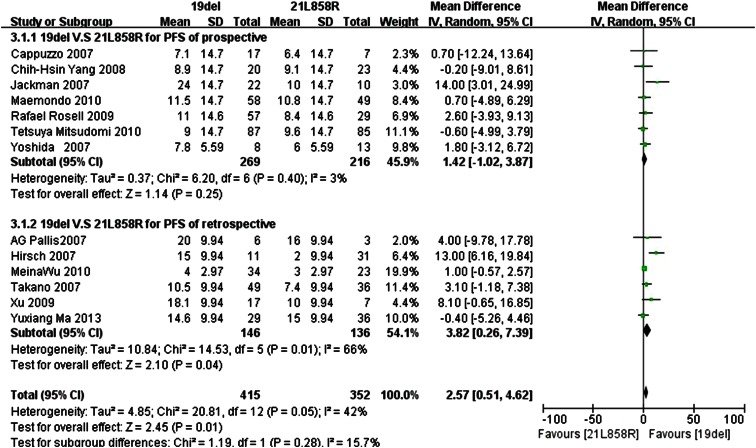
Forest plot of PFS among patients with EGFR 19del or 21L858R mutations with maximum SD. The *squares* and *horizontal lines* correspond to the study-specific MD and 95 % CI. The area of the *squares* reflects the weight. The *diamond* represents the summary RR and 95 % CI

Similarly, we repeated the analysis with the standard deviation set to the maximum value of 14.7 for the other four prospective trials, and the maximum value 9.94 for the other for retrospective trials. Because the *I* ^2^ statistic in the fixed effects model demonstrated statistically significant heterogeneity in the results (*I* ^2^ 42 %;* P* = 0.05), a random effects model was used to pool the mean difference for the included studies. As Fig. 4 demonstrates, patients with the exon 19del mutation had a statistically significant longer PFS than patients with the exon 21L858R mutation (MD 2.57; 95 % CI 0.51–4.62; *P* = 0.01). As the subgroup analysis shows, in the prospective trials, subgroup patients with exon 19del mutation had a longer PFS than patients with exon 21L858R mutations, but the difference was not statistically significant (MD 1.42; 95 % CI −1.02 to 3.87; *P* = 0.25; *I* ^2^ = 3 %, random effects model). However, patients with the exon 19del mutation had a statistically significant longer PFS than patients with the exon 21L858R mutations in the retrospective trials subgroup (MD 3.82, 95 % CI 0.26–7.39; *P* = 0.04; *I* ^2^ = 66 %, random effects model).

### Overall survival (OS)

**Fig. 5:**
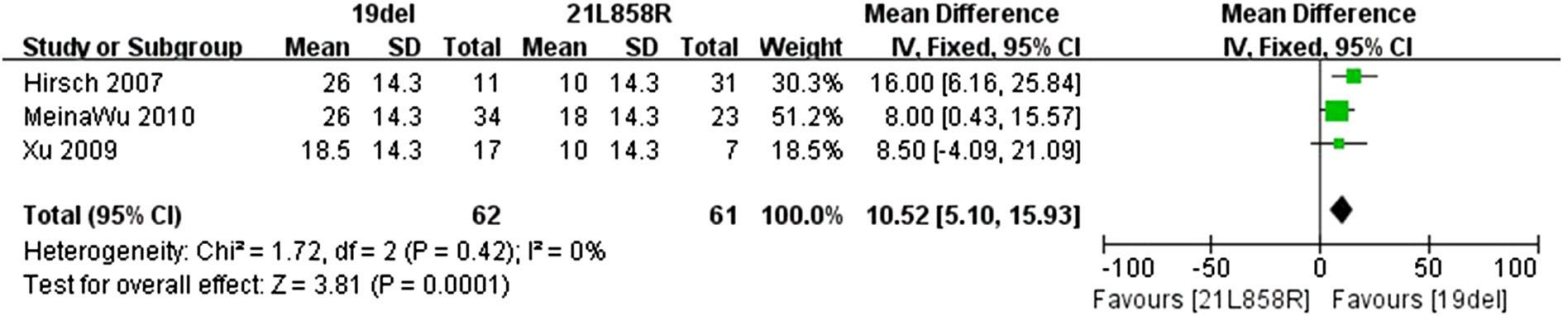
Forest plot of OS among patients with EGFR 19del or 21L858R mutations. The *squares* and *horizontal lines* correspond to the study-specific MD and 95 % CI. The area of the *squares* reflects the weight

Data for the OS were available in three of the retrospective trials. The standard deviation for the OS could be obtained in Xu’s trial. But it was necessary to set the value for the other two trials. The *I* ^2^ statistic in the fixed effects model did not demonstrate significant heterogeneity in the results (*I* ^2^ 0 %; *P* = 0.42), so the fixed effects model was used to pool the mean difference for the included studies. As shown in Fig. 5, patients with exon 19del mutation had a statistically significant longer OS than patients with exon 21L858R mutation (MD 10.52; 95 % CI: 5.10–15.93; *P* = 0.0001).

### Publication bias

**Fig. 6:**
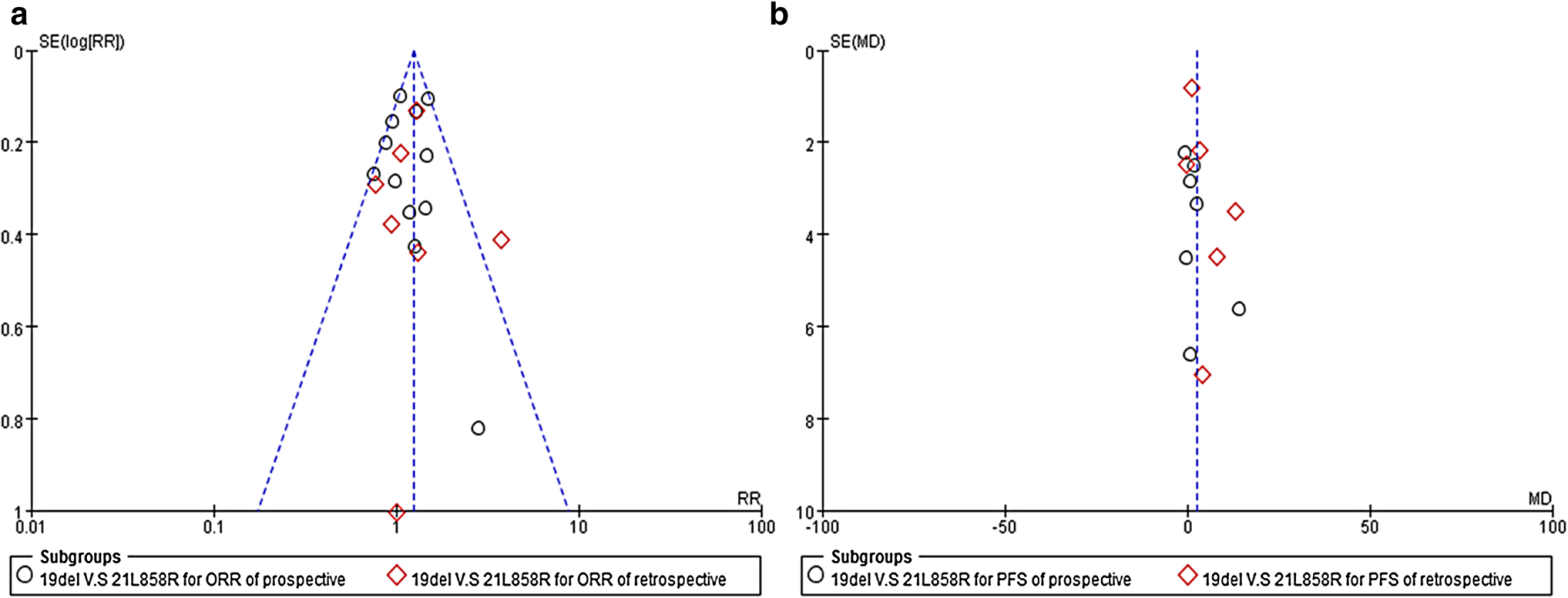
**a** Funnel plot of the relative ORR; **b** funnel plot of the relative PFS

The funnel plot for the overall pooled analysis of the association between subtypes of EGFR mutations, and response (Fig. 6a) revealed no evidence of publication bias, with a symmetrical distribution of study results around the pooled measurement of effect. Moreover, the funnel plot for the overall pooled analysis of the association between types of EGFR mutations, and PFS (Fig. 6b) also revealed no evidence of publication bias. The mean of the pooled effect was positive which indicated a potential publication bias in favor of more positive studies. However, the evaluation of publication bias using the funnel plot approach was somewhat limited by the small number of studies identified for inclusion in the pooled analyses.

### Variance analysis

We, respectively, compared the PFS of 767 patients and the OS of 123 patients through variance analysis, using SPSS software.

As shown in Table 2, the PFS data are normally distributed (*P* > 0.05). The average PFS was 11.1 months in patients with the exon 19del mutation and 8.7 months in patients with the exon 21L858R mutation, this difference is statistically significant (*P* < 0.05). As the results of the rank and *U* test show, the overall survival data had a non-normal distribution(*P* < 0.001), and the average overall survival in patients with the exon 19del mutation was 23.9 and 13.0 months for patients with the exon 21L858R mutation, this difference is also statistically significant.

**Table 2:** Variance analysis for the progression-free survival data, and rank and sum test—nonparametric (*U* test) for the overall survival data

	19del	21L858R	*F* (*P* value)	*P* value	*P* value (*U* test)
PFS (month)	11.1	8.7	3.673 (*P* > 0.05)	0.000	
OS (month)	23.9	13.0	11.283 (*P* < 0.001)		*P* < 0.05

## Discussion

The correlation of EGFR TKI efficacy and EGFR mutations subtypes is the important factor in most of the large phase III clinical trials in NSCLC patients with EGFR sensitive mutations, but the results are difficult to combine. According to analyses of the five US and European clinical trials that assessed first-line TKI treatment, patients with the exon 19 deletion have a significantly longer progression-free and overall survival time than patients with L858R (30.8 vs. 14.8 months; *P* < 0.0001) (Morita et al. 2009). Studies on North American patients indicated that those with deletions of exon 19 in the EGFR have a better response rate, progression-free survival and overall survival after EGFR tyrosine kinase inhibitor treatment than do those with the L858R mutation in exon 21 (Jackman et al. 2006; Riely et al. 2006). A Spanish study led by Rosell also indicated that patients with the 19del mutation had a better response than those with the L858R mutation (odds ratio, 3.08; 95 % CI 1.63–5.81; *P* = 0.001); however, there was no significant difference in the progression-free survival time with respect to mutation type (Rosell et al. 2009). The results of the IPASS study showed that compared with the patients with the L858R mutation, the PFS of patients with the 19del mutation was not statistically different (HR 0.55; 95 % CI 0.35–0.87); however, the ORR was higher (84.8 %), but the difference was not statistically significant (OR 1.41; 95 % CI 0.65–3.05) (Fukuoka et al. 2011). The same results were acquired from WJTOG3405 and EURTAC studies (Mitsudomi et al. 2010). Differences in the results of these studies may be related to the sample count. For this reason, we systematically evaluated the differences between the two common EGFR mutations and the efficacy of EGFR TKIs.

Our analysis adds to our understanding of the importance of the influence of EGFR mutations on the efficacy of EGFR TKI. The results of this meta-analysis comparing the difference in efficacy of EGFR TKI in advanced NSCLC for the EGFR 19del and 21L858R mutations, confirms that the EGFR 19del mutations when treated with EGFR TKI show a better response rate and progression-delaying effect than the EGFR 21L858R mutations.

The results of this pooled analysis highlight the idea that the exon 19del mutation is a better indicator of strong efficacy in EGFR TKI treatment, which is an improvement over findings in patients with lung cancer that have been reported previously. These results also contribute to evidence-based medicine in clinical work. Screening for EGFR mutations is warranted and should be performed before beginning therapy with EGFR TKIs, since the exon 19del mutation implies a better outcome than the L858R mutation.

As our meta-analysis results have shown, the subgroup analysis showed that there was no statistically significant difference in PFS, the possible reason may be that the small sample size results in the lack of statistical significance. The number of subgroups in the included literature is too few, totaling only seven prospective studies and six retrospective studies. Moreover, only three cases in the literature were included in the pooled analysis of OS, so the sample number was low. Unfortunately, most of the prospective and retrospective studies failed to obtain the overall survival data, so the evaluation of the difference in OS needs further verification.

We estimated the standard deviation in order to evaluate the data of progression-free survival and overall survival for studies with a poor level of evidence-based medicine. Toward this end, we compared the difference in the mean for the two types of EGFR mutations and overall survival in patients using SPSS software. The results showed that progression-free survival and overall survival in patients with the exon 19del mutation were significantly longer than patients with the exon 21L858R mutation, which is in accordance with the results of the meta-analysis.

Comprehensive literature was included in this pooled analysis which contains both prospective and retrospective studies, and the number of objects was determined to be sufficiently large. The ORR, progression-free survival and overall survival were all selected to evaluate the difference in EGFR TKI efficacy between exon 19del and 21L858R mutations; our evaluation indicators are comprehensive thereby. However, our evaluation has the following limitations; firstly, due to lack of research about the relationship between EGFR mutation type and the efficacy of EGFR TKI, all the data came from their subgroups, which could lead to the data being incomplete. Secondly, the instability problems of the retrospective studies also influenced the quality of the system analysis.

## Conclusion

The results of this system evaluation suggest that in III-IV NSCLC patients with EGFR mutations, patients with the exon 19 deletion have a better response rate, and a significantly longer progression-free survival and overall survival than patients with the L858R mutation. Hence, the type of EGFR mutation has predictive value for EGFR TKI efficacy, and patients with the exon 19del mutation show a more prominent preponderance in efficacy for EGFR TKIs. The difference may be related to the different biological activities of EGFR mutations. The mechanism is not entirely clear, so further exploration is needed at the molecular level in the future.
